# Highlight report: Necrosis-apoptosis conundrum of hepatocytes: mode of hepatocyte death after acetaminophen intoxication

**DOI:** 10.17179/excli2018-2013

**Published:** 2018-12-19

**Authors:** Ahmed Ghallab

**Affiliations:** 1 Forensic Medicine and Toxicology Department, Faculty of Veterinary Medicine, South Valley University, Qena, Egypt

## ⁯⁯⁯

Recently, Huo Du from Hartmut Jaesches's group at University of Kansas published an outstanding study about the mode of hepatocyte killing by acetaminophen and demonstrate that the typical necrotic cell death may switch to secondary apoptosis after specific interventions (Du et al., 2018[7]).

Acetaminophen (APAP) is responsible for more than 70.000 hospitalizations per year and approximately 50 % of acute liver failure cases (Budnitz et al., 2011[5]; Manthripragada et al., 2011[25]). Cell killing by APAP is a consequence of cytochrome P450-mediated formation of the reactive N-acetyl-p-benzoquinone imine (NAPQI) that binds to proteins and glutathione (Mc Gill and Jaeschke, 2013[27], 2015[26]; Nelson, 1989[29]; Xie et al., 2015[38]). It is well accepted that APAP kills hepatocytes predominantly by necrosis rather than by apoptosis (Bajt et al., 2004[3]; Gujral et al., 2002[16]; Jaeschke et al., 2011[21]; McGill et al., 2011[28]). This phenomenon has been observed *in vivo *and* in vitro.* The preference for necrosis remains difficult to understand, because some mechanisms considered specific for apoptosis are still induced by APAP, such as mitochondrial translocation of bax and release of cytochrome C, but nevertheless do not lead to apoptotic phenotype (Du et al. 2016[6]; Adams et al., 2001[1]; Knight and Jaeschke, 2002[23]; Bajt et al., 2008[2]).

In their present study, Du and colleagues came much closer to an explanation of this conundrum (Du et al., 2018[7]). They used the mitochondria-targeted superoxide dismutase mimetic Mito-tempo in mice intoxicated with a hepatotoxic dose of 300 mg/kg APAP. As expected, Mito-tempo reduced APAP induced necrosis and led to an overall protection against hepatotoxicity. However, some hepatocytes switched to a clearly apoptotic phenotype as evidenced by morphology, TUNEL positivity and caspase activation, which was not observed after APAP intoxication without Mito-tempo administration (Du et al., 2018[7]). In an elegant series of experiments using RIP3 knockout mice and decreasing RIP3 protein levels by a RIP3-morpholino, the authors demonstrate that the effect of Mito-tempo is due to inhibition of RIP3. In conclusion, the necrosis-apoptosis conundrum of hepatocytes is due to RIP3 kinase, which tips the balance to necrosis, while its inhibition switches cell death to necrosis.

Currently, hepatotoxicity is a major research focus, because drug induced liver injury represents a frequent cause of drug withdrawal from the market (Godoy et al., 2013[11], 2016[12]; Hewitt et al., 2007[20]; Reif et al., 2017[30]).

Numerous studies aim at a better understanding of the molecular and pathophysiological mechanisms of hepatotoxicity (Hassan, 2016[19]; Stöber, 2016[35]; Sezgin et al., 2018[33]; Jansen et al., 2017[22]; Vartak et al., 2016[37]; Ghallab et al., 2016[10]; Bolt, 2017[4]; Schenk et al., 2017[32]; Thiel et al., 2015[36]; Hammad et al., 2014[18]). A frequently applied strategy in toxicology is to construct 'adverse outcome pathways' (Leist et al., 2017[24]; Rodrigues et al., 2018[31]) aiming for possibilities to study hepatoxicity *in vitro *and* in silico *(Ghallab, 2017[9]; Hammad, 2013[17]; Grinberg et al., 2014[14], 2018[13]; Gu et al., 2018[15]; Shinde et al., 2015[34]; Frey et al., 2014[8]).

However, this strategy is hampered by the fact that so many aspects of hepatotoxicity *in vivo* remain elusive, even for a compound as intensively studied as APAP. In conclusion, Huo Du and colleagues are to be congratulated that they unraveled a mystery that confused toxicologists since decades.
